# 16S rRNA Gene Metabarcoding Indicates Species-Characteristic Microbiomes in Deep-Sea Benthic Foraminifera

**DOI:** 10.3389/fmicb.2021.694406

**Published:** 2021-07-27

**Authors:** Iines S. Salonen, Panagiota-Myrsini Chronopoulou, Hidetaka Nomaki, Dewi Langlet, Masashi Tsuchiya, Karoliina A. Koho

**Affiliations:** ^1^Ecosystems and Environment Research Program, University of Helsinki, Helsinki, Finland; ^2^SUGAR, X-star, Japan Agency of Marine-Earth Science and Technology (JAMSTEC), Yokosuka, Japan; ^3^UMR 8187 - LOG - Laboratoire d’Océanologie et de Géosciences, Université de Lille - CNRS, Université du Littoral Côte d’Opale, Station Marine de Wimereux, Lille, France; ^4^Evolution, Cell Biology, and Symbiosis Unit, Okinawa Institute of Science and Technology, Okinawa, Japan; ^5^Research Institute for Global Change (RIGC), Japan Agency of Marine-Earth Science and Technology (JAMSTEC), Yokosuka, Japan

**Keywords:** foraminifera, unicellular eukaryotes, sediment, deep sea, endobionts, metabarcoding

## Abstract

Foraminifera are unicellular eukaryotes that are an integral part of benthic fauna in many marine ecosystems, including the deep sea, with direct impacts on benthic biogeochemical cycles. In these systems, different foraminiferal species are known to have a distinct vertical distribution, i.e., microhabitat preference, which is tightly linked to the physico-chemical zonation of the sediment. Hence, foraminifera are well-adapted to thrive in various conditions, even under anoxia. However, despite the ecological and biogeochemical significance of foraminifera, their ecology remains poorly understood. This is especially true in terms of the composition and diversity of their microbiome, although foraminifera are known to harbor diverse endobionts, which may have a significant meaning to each species’ survival strategy. In this study, we used 16S rRNA gene metabarcoding to investigate the microbiomes of five different deep-sea benthic foraminiferal species representing differing microhabitat preferences. The microbiomes of these species were compared intra- and inter-specifically, as well as with the surrounding sediment bacterial community. Our analysis indicated that each species was characterized with a distinct, statistically different microbiome that also differed from the surrounding sediment community in terms of diversity and dominant bacterial groups. We were also able to distinguish specific bacterial groups that seemed to be strongly associated with particular foraminiferal species, such as the family *Marinilabiliaceae* for *Chilostomella ovoidea* and the family *Hyphomicrobiaceae* for *Bulimina subornata* and *Bulimina striata*. The presence of bacterial groups that are tightly associated to a certain foraminiferal species implies that there may exist unique, potentially symbiotic relationships between foraminifera and bacteria that have been previously overlooked. Furthermore, the foraminifera contained chloroplast reads originating from different sources, likely reflecting trophic preferences and ecological characteristics of the different species. This study demonstrates the potential of 16S rRNA gene metabarcoding in resolving the microbiome composition and diversity of eukaryotic unicellular organisms, providing unique *in situ* insights into enigmatic deep-sea ecosystems.

## Introduction

Benthic foraminifera are single-celled eukaryotes, widespread in global oceans and particularly abundant in deep-sea sediments, accounting for up to 50% or more of the total eukaryotic biomass in places (Snider et al., 1984; Gooday et al., 1992). They are important consumers of phytodetritus (Gooday, 1988; Ohga and Kitazato, 1997; Moodley et al., 2002), experimentally shown to consume carbon faster than metazoans (Nomaki et al., 2005). Part of their ecological success in the benthic environment may be attributed to their ability to thrive in the low-oxygen environments (e.g., Moodley and Hess, 1992; Bernhard, 1993; Jorissen et al., 1995; Moodley et al., 1997; Langlet et al., 2013). Adaptations to hypoxia may include, for example, the wide-spread ability of foraminifera to accumulate and respire nitrate (Risgaard-Petersen et al., 2006; Piña-Ochoa et al., 2009), as well as their capability to reduce metabolism, suspend reproduction, and enter a state of dormancy (LeKieffre et al., 2017; Koho et al., 2018; Richirt et al., 2020).

A yet unresolved link in deep-sea foraminiferal ecology and environmental adaptation is the composition and role of their associated microbiomes. Benthic foraminifera are known to develop endobiotic relationships and close associations with prokaryotes (e.g., Bernhard, 2003; Bernhard et al., 2006, 2018), which may include denitrifiers (Bernhard et al., 2011), sulphur cycle-related bacteria (Tsuchiya et al., 2015; Salonen et al., 2019), *Cyanobacteria* (Prazeres et al., 2017), or even methanotrophs (Bernhard and Panieri, 2018). The endobiotic relationships between foraminifera and bacteria have been hypothesized to provide the host with metabolic flexibility, alternative carbon assimilation pathways, and enhanced nutrient/organic compound cycling, enabling them to not only survive but also flourish in the harsh benthic environment (Bernhard et al., 2011; Bird et al., 2017; Prazeres et al., 2017; Tsuchiya et al., 2018; Salonen et al., 2019). Despite the ecological importance of microbiome in foraminiferal adaptation, very little is known of its prevalence, significance, and evolution. Some studies indicate that the prokaryotic composition of the microbiome may be flexible and display a considerable amount of local variation (Prazeres et al., 2017) whereas evidence of species-specific microbiomes have also been observed in shallow intertidal areas with no indication of spatial variability (Salonen et al., 2019).

Foraminiferal microbiome may also be directly linked to their microhabitat preferences (Fontanier et al., 2005). In sediments, foraminifera display species-specific preferences in their vertical distribution, which may depend on the geochemical zonation of the sediment and the availability of oxygen, other electron acceptors (e.g., nitrate), and organic matter (e.g., Corliss, 1985; Jorissen et al., 1995; Fontanier et al., 2002; Koho and Piña-Ochoa, 2012). Microhabitat preferences are typically reflected in foraminifera’s trophic behavior and survival strategies (e.g., Kitazato, 1988; Tsuchiya et al., 2018). For example, some deep-sea epifaunal and shallow-infaunal species, living above or within the surface sediments, respectively, have been suggested to entirely depend on an opportunistic lifestyle specialized in rapid consumption of phytodetritus in response to phytoplankton blooms occurring in the ocean surface (Gooday, 1988). They also typically migrate faster in sediments than the deep-dwellers (Kitazato, 1988; Ernst et al., 2002). In contrast, deep infaunal species, which can live below oxygen penetration depth, react slower to the fresh organic matter inputs (Nomaki et al., 2005, 2006). This may be due to their suspected longer life cycle, during which they are likely to rely on deposit-feeding and consumption of degraded organic matter instead of fresh phytodetritus (Goldstein and Corliss, 1994; Ohga and Kitazato, 1997; Schmiedl et al., 2004). However, the exact feeding preferences of foraminifera, especially on species level, are poorly resolved due to the difficulty of examining them *in situ*. New molecular approaches, such as metabarcoding, can provide new insights on foraminiferal ecology and their feeding preferences (Chronopoulou et al., 2019).

In marine science, the research on microbiomes has rapidly increased during the last decade, but host-associated microbiome research is still mainly focused on multicellular and larger animals, such as sponges and corals, instead of single-celled organisms (Trevathan-Tackett et al., 2019). In the case of foraminifera, previous studies on their intracellular bacterial communities have been mainly based on transmission electron microscopy (TEM) observations (e.g., Bernhard et al., 2018; Koho et al., 2018). However, the current molecular ecology tools, such as DNA metabarcoding, offer new, efficient ways to investigate the diversity and composition of microbiomes of the unicellular foraminifera (e.g., Bird et al., 2017; Prazeres et al., 2017; Salonen et al., 2019).

In this study, we applied the definition of microbiome by Trevathan-Tackett et al. (2019), stating that microbiome is a consortium of intracellular bacteria derived from genetic material, to investigate the microbiome of deep-sea foraminifera with variable microhabitat distributions. We utilized a DNA metabarcoding approach based on 16S rRNA gene to investigate the microbiome characteristics of deep-sea foraminifera in terms of intracellular bacterial and chloroplast composition. Comparison was also made with the surrounding bacterial sediment community to detect and identify bacteria that are enriched in relative abundance (RA) inside the foraminifera. The bacterial composition of the foraminiferal microbiome is likely reflecting the ecological characteristics of the given foraminiferal species, and therefore, the overall aim of the study was to gain insights into individual ecological strategies of the studied foraminiferal species and identify bacteria-foraminifera interactions that may be endobiotic in nature. In addition, the foraminiferal chloroplast composition was used to identify potential differences in trophic strategies.

## Materials and Methods

### Sediment Sampling

The sampling was carried out onboard the research vessel R/V *Seiyo Maru* in early June 2017 at Sagami Bay, Japan (35°02ꞌ52ꞌꞌN 139°19ꞌ46ꞌꞌE). Samples were taken with a mini-multiple corer equipped with four cores (inner diameter 8.2 cm), allowing the collection of undisturbed sediments. Altogether, three casts were deployed and eight cores from 734 to 735 m water depth were collected.

Pore water sampling (see below for details) from two cores (SB4 and SB5) was carried out immediately after sampling, on board the research vessel (Table 1). Afterwards all cores were kept cool and transported into the JAMSTEC laboratory within 8 h of sampling for core slicing and further processing. Cores SB4 and SB5 were sliced for Rose Bengal staining of foraminifera (see Supplementary Additional Figure 1). Five cores (SB1–SB3, X2, and X3) were used for the collection of living benthic foraminifera of which three (SB1–SB3) were also sampled for sediment bacterial community (see more details below). A core (SB6) was also used for dissolved oxygen profiling. In addition, a ninth core (SB19) was collected from the same site at Sagami Bay in September 2019 during the R/V *Kaimei* KM19-07 cruise and was used for additional oxygen profiling and pore water nutrients (nitrate, nitrite, and ammonium) analysis (Table 1).

**Table 1:** Sediment cores used in this study and their processing.

Core name	Purpose					
	Picking of foraminifera	Sediment bacterial community	Pore water Mn, Fe	Pore water nutrients	Dissolved oxygen	Core processing
SB1	X	X				JAMSTEC
SB2	X	X				JAMSTEC
SB3	X	X				JAMSTEC
SB4			X			on board/JAMSTEC
SB5			X			on board/JAMSTEC
SB6					X	JAMSTEC
X2^*^	X					JAMSTEC
X3^*^	X					JAMSTEC
SB19^°^				X	X	on board

* Only the top 0–1 cm of the core was used.

° Year 2019.

### Pore Water Sampling and Analyses

Pore water was extracted on board the research vessel using Rhizons™ (Rhizosphere Research Products B.V., The Netherlands). Prior to the deployment, cores (SB4, SB5; Table 1) with predrilled holes were covered using water resistant tape. Immediately after retrieving the cores, the tape was removed, and Rhizons™ with connected 10 ml plastic syringes were used to extract pore water with 1 cm intervals starting from 0.5 cm sediment depth down to 10 cm. After, the samples were acidified with suprapur 1 M HNO_3_ (10 μl per 1 ml of sample) and stored for elemental analyses (namely for analyses of iron and manganese) with inductively coupled plasma-optical emission spectrometry (ICP-OES) at the Geolab of Utrecht University, the Netherlands.

Core SB6 (Table 1) was used for oxygen profiling. Five oxygen profiles were retrieved using 100 μm tip oxygen microsensor (Unisense, Denmark) with a 100 μm vertical resolution. The oxygen microsensor was two-point calibrated in an anoxic sodium-ascorbate solution and a saturated solution of sea water. Overlying water temperature was 11–14°C.

During the KM19-07 cruise in 2019, onboard analyses of a core (SB19; Table 1) for both dissolved pore water oxygen and nutrients (nitrite, nitrate, and ammonium) were carried out as described in Langlet et al. (2020). In brief, oxygen micro-profiling was carried out in a cold room at 4°C immediately after sediment sampling. The overlying water for nutrient analyses was sampled prior to the oxygen profiling. After the oxygen profiling, sediments were sliced at every 1 or 2 cm thickness and centrifuged at 2,600 × *g* for 5 min. The supernatant was sampled for porewater, filtered together with the overlaying water sample over a 0.45 μm syringe filter, and subsequently measured for dissolved nitrogen species with a continuous-flow analyzer (BL-Tech QUAATRO 2-HR system).

### Sediment Processing and Extraction of Living Foraminifera

At JAMSTEC laboratory, three of the cores (cores SB1, SB2, and SB3; Table 1) were sliced down to 5 cm depth, first 2 cm with 0.5 cm intervals, and then with 1 cm intervals (Supplementary Additional Figure 2). From each sediment slice, a subsample of approximately 1–2 g (wet weight) of sediment was taken with a sterile plastic spatula and placed into a sterile vial for the analysis of the surrounding sediment bacterial community. The bacterial samples were immediately frozen in liquid nitrogen and stored in −20°C until DNA extraction. Two additional cores (X2 and X3) were used to retrieve living foraminifera from which only a top slice of 0–1 cm was examined (Table 1; Supplementary Additional Figure 2).

The sediment slices for foraminifera were subsequently sieved using filtered natural seawater through 150 μm mesh, and living foraminifera were picked in a chilled-petri dish under binocular microscope. Only specimens leaving a displacement track on a thin layer of sediment and confirmed active were selected, as described in Koho et al. (2018). After morphological identification under the stereomicroscope, each specimen was thoroughly washed three times with sterile artificial nitrate-free sea water (ASW, prepared with 35 g of RedSea salt per litter of MilliQ ultrapure water, and autoclaved before use).

The foraminifera selected for microbiome analysis were placed into a sterile vial filled with RNA*later* solution, which dissolves the calcite shell of foraminifera while preserving the DNA. The samples were stored in RNA*later* solution in +8°C until DNA extraction (see Bird et al., 2017). Before extraction, the naked foraminiferal cells were again washed three times with sterile ASW to obviate any remaining shell material or residual RNA*later* solution from hampering the downstream analysis (Bird et al., 2017; Salonen et al., 2019). By dissolving the shell and performing repeated washing prior and after that, we ensured that the extracted bacterial community was indeed intracellular and not shell associated.

The 16S rRNA gene of altogether 50 specimens was successfully (see Supplementary Additional Table 1) sequenced for this study: four *Bulimina striata*, 18 *Bulimina subornata*, 16 *Chilostomella ovoidea*, 11 *Globobulimina pacifica*, and two *Nonionella labradorica*. The five species studied here have contrasting microhabitat preferences, as *B. subornata* and *B. striata* are shallow-infaunal species (Kitazato, 1989; Supplementary Additional Figure 1), *N. labradorica* is a shallow- and intermediate-infaunal species (Kitazato, 1989; Fontanier et al., 2014; Supplementary Additional Figure 1), and *C. ovoidea* and *G. pacifica* are deep-infaunal species (Corliss, 1985; Jorissen et al., 1995; Ohga and Kitazato, 1997; Supplementary Additional Figure 1).

### Denitrification and Nitrate Pool Measurements

After the foraminifera were identified and cleaned, 5–11 individuals of the same species were pooled together to test denitrification activity and determine intracellular nitrate quantity. Nitrate respiration rates were then determined based on Fick’s first law of diffusion from steady-state N_2_O profiles measured with a N_2_O micro electrode (Andersen et al., 2001) after acetylene inhibition of N_2_O reduction (Smith et al., 1978; Risgaard-Petersen et al., 2006; Høgslund et al., 2008) in anoxic and nitrate free ASW at 11°C.

Specimens selected for intracellular nitrate determination were placed in 1 ml centrifugation microtubes and frozen at −80°C to break the foraminiferal cell and conserve the samples until further nutrient content analysis by following Nomaki et al. (2015) and Langlet et al. (2020). Nitrate and nitrite extraction from the foraminiferal cells was achieved by freezing at −80°C, and the frozen cells were then dissolved in 200 μl of MilliQ water and homogenized in the microtube using a plastic pestle. NO_3_^−^ + NO_2_^−^ was determined by the vanadium (III) chloride reduction method adapted from Doane and Horwáth (2003) where 160 μl of the aliquot was mixed with 20 μl of nitrate reductant (8 g of VCl_3_ per liter of 0.6 mol/l HCl) and 20 μl of color reagent (prepared from 2 g sulfanilamine and 100 mg n-1-naphthyl ethylenediamine dihydrochloride per liter of 0.6 mol/l HCl) and heated at 60°C for 2 h. Calibration was performed using 0.1–50 μmol/l NO_3_^−^ + NO_2_^−^ standards prepared with KNO_3_ and MilliQ water. The NO_3_^−^ + NO_2_^−^ concentration was determined from the absorbance at a wavelength of 540nm with a UV–VIS spectrophotometer (UV-1800, Shimadzu corp.) and normalized to the number of individuals to calculate individual NO_3_^−^ + NO_2_^−^ content (in pmol per individual).

### DNA Extraction, Amplification, and Illumina MiSeq Sequencing

The DNA from within each foraminiferal specimen was extracted within 2 months of collection using the DOC method (Holzmann and Pawlowski, 1996). Sediment DNA from the three replicates was extracted using the DNeasy PowerSoil® DNA Isolation Kit (Qiagen, Germany). The DNA from foraminifera and sediment was amplified using universal bacterial primers 27F (5ꞌAGAGTTTGATCMTGGCTCAG) and 519R (5ꞌGTATTACCGCGGCTGCTG) targeting the variable regions V1–V3 of the 16S rRNA gene, in a similar way as described in Salonen et al. (2019). Duplicate PCR reactions were performed and pooled in equal volumes, in order to ensure adequate amplicon volume for Illumina MiSeq library preparations and minimize PCR amplification bias. All amplicons were checked with agarose gel electrophoresis. Sequencing was carried out on the Illumina MiSeq platform in the laboratory of DNA sequencing and Genomics in the Institute of Biotechnology, at the University of Helsinki. Prior to sequencing, the amplicons were purified and multiplexed as described in Salava et al. (2017). Barcodes for later sample de-multiplexing were selected using BARCOSEL (Somervuo et al., 2018).

As single-cell DNA extractions are susceptible to contamination, careful measures were taken to monitor and exclude any bacterial signal non-related to foraminifera. Blank samples were taken for all the steps and reagents involved in the foraminiferal processing, including RNA*later* (two blanks), ASW used for cleaning of the foraminifera (three blanks) and the DOC extraction buffer (one blank sample). Negative controls were routinely checked in the PCR step. Regardless of whether the blank samples produced a visible band on the agarose gel or not, they were sequenced and analyzed alongside the rest of the samples. Additionally, one negative control that produced a faint band on the agarose gel was also sequenced and analyzed. The blank and negative control samples were of low sequencing depth and resulted in a distinct community where the most abundant bacterial groups included *Propionibacterium*, *Sphingomonas*, *Corynebacterium*, and *Pseudomonas*, which are common contaminants in DNA extraction kits and laboratory reagents (Salter et al., 2014). In addition, we observed a *Halomonas* contamination particularly associated with the ASW blank samples. The operational taxonomic units (OTUs) associated with the blank samples and negative control (in total 1.4% of all reads) were bioinformatically removed from the final dataset. In addition, mitochondrial sequence reads and unclassified OTUs were removed from the dataset.

Illumina MiSeq sequencing produced 4,789,674 sequence reads in the sediment dataset (23 sediment samples) and 9,660,770 in the foraminiferal dataset (50 specimens in total). Trimming, quality-checking, and removal on chimeric sequences reduced the datasets to 2,558,292 and 5,534,414 reads for sediment and foraminifera, respectively. These reads were clustered into 449,494 and 141,217 OTUs. To avoid overestimating diversity, the OTU number was further reduced by plotting the total number of reads against estimated cutoffs (see Chronopoulou et al., 2019; Salonen et al., 2019). A cutoff of 20 OTUs per sample reduced the number of reads to 1,883,492 (95.9% of original reads) in the sediment dataset, consisting of 9,056 unique OTUs in total. In the foraminiferal data, a cutoff of five OTUs was used, which reduced the amount of reads to 2,834,713 (95.8% of original reads) and the number of OTUs to 8,489 in total. The number of OTUs and reads per individual samples are reported in the Supplementary Additional Table 2.

### Full-Length 16S rRNA Gene Sequencing

In addition, DNA extracts from four *B. subornata*, one *B. striata*, one *G. pacifica*, two *C. ovoidea*, and one sediment sample (0–0.5 cm sediment depth, core SB1) were amplified with primers 27F (5ꞌ-AGAGTTTGATCMTGGCTCAG-3ꞌ) and 1492R (5ꞌ-GGTTACCTTGTTACGACTT-3ꞌ) to obtain full-length 16S rRNA gene sequences (Lane, 1991). A fragment of approximately 1,465 bp was sequenced with PacBio RS II sequencing instrument (Pacific Biosciences) in the Institute of Biotechnology, University of Helsinki, Finland. These long 16S rRNA gene sequences were analyzed in a similar way to shorter MiSeq reads, except adjusting the maximum length to 1,465 bp. The full-length sequencing of the 16S rRNA gene produced in total 20,480 sequences that formed 3,298 OTUs.

### Bioinformatical and Statistical Analysis

Raw sequences were de-multiplexed based on barcodes. Then, MiSeq overhangs, primers, and barcodes were removed, and sequences were quality-checked, trimmed (maximum length adjusted to 550 bp), and aligned using Mothur, following the standard operating procedure (version 1.41.1; Schloss et al., 2009). Chimeric sequences were removed in Mothur using the UCHIME algorithm (Edgar et al., 2011). Taxonomy was assigned against the SILVA reference database (release 132). OTU tables created in Mothur were analyzed in R (version 3.6.3; R Core Team 2020) using the package phyloseq (version 1.30.0; McMurdie and Holmes, 2013). Non-metric multidimensional scaling (nMDS) was used to visualize the separation of foraminiferal species and the five most common intracellular bacterial groups in the ordination space. Permutational multivariate ANOVA (PERMANOVA) test using the adonis2 function in Vegan (Oksanen et al., 2015) was used to analyze the significant variables (e.g., species and sediment depth) influencing the sediment bacterial community and the foraminiferal microbiome. For the nMDS, Hellinger transformed (Legendre and Gallagher, 2001) data were used. Alpha diversity estimates are dependent on singletons and doubletons, and therefore, untrimmed and non-normalized data (as recommended by McMurdie and Holmes, 2014) were used to calculate Shannon diversity (*Hꞌ*) and Pielou’s evenness (*J*ꞌ) indices in both sediment and foraminiferal datasets using the Vegan package (version 2.5-6; Oksanen et al., 2015).

### Phylogenetic Analysis

Closest relatives for the phylogenetic trees were obtained using a BLAST search against the NCBI GenBank database (Altschul et al., 1990). Representative sequences of the most common chloroplast OTUs, including *Cyanobacteria*, in foraminifera (24 OTUs in total) and sediment (six OTUs in total) were aligned with their closest matches from NCBI GenBank using the MUSCLE algorithm (v. 3.8.31; Edgar, 2004). Aligned sequences were manually checked, and Maximum likelihood phylogenetic trees were constructed with MEGA7 (version 7.0.26; Kumar et al., 2016).

## Results

### Porewater Geochemistry

**Figure 1:**
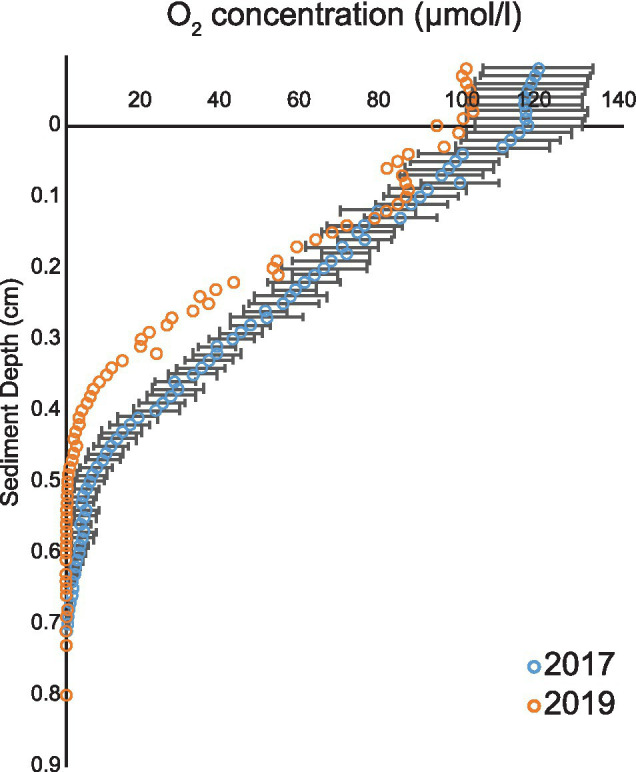
Oxygen profiles from years 2017 and 2019. Average (blue open circles) and SEM (black line) of five O_2_ profiles during 2017 and 2019 cruises (orange open circles).

In the 2017 measurement, the overlying water oxygen (O_2_) concentration ranged from 100 to 130 μmol/l and O_2_ penetration depth in sediment varied from 0.52 to 0.85 cm (Figure 1). The results were consistent with measurements of 2019 when the overlying water O_2_ concentration was 100 μmol/l and the penetration depth 0.6 cm (Figure 1). As the measured O_2_ conditions in the 2 years were similar (Figure 1), an assumption was made that the pore water ammonium (NH_4_^+^), nitrite (NO_2_^−^), and nitrate (NO_3_^−^) concentrations in year 2017 resembled the 2019 situation.

**Figure 2:**
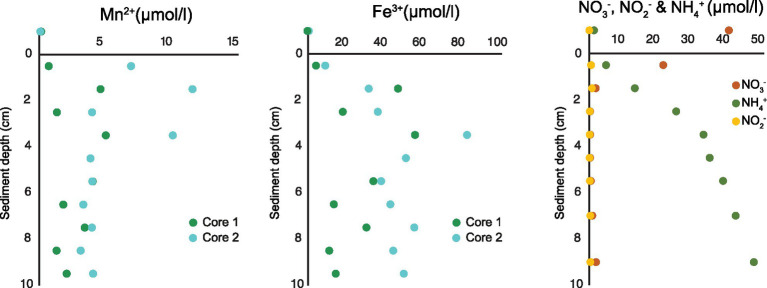
Porewater profiles of manganese and iron (2017) and nitrate, nitrite, and ammonium (2019) in 0–10 cm sediment depth.

Below oxygen and nitrate penetration depth manganese (Mn^2+^) and iron (Fe^3+^) reduction was detected. The concentration of Mn^2+^ reached up to 11.5 μmol/l at approximately 1.5 cm sediment depth and concentration of Fe^3+^ up to 80 μmol/l at approximately 3.5 cm sediment depth (Figure 2). Based on measurements from 2019 (Table 1), a NO_3_^−^ reduction zone was present in the sediment down to approximately 1.5 cm depth (Figure 2), which is consistent with the Mn reduction taking place at similar sediment depths. The concentration of NH_4_^+^ increased steadily below the O_2_ penetration depth, reaching 47 μmol/l at 10 cm sediment depth (Figure 2).

### Intracellular Nitrate Content and Nitrate Respiration

Intracellular nitrate + nitrite pool was measured for three species, including *B. subornata*, *C. ovoidea*, and *G. pacifica*. The largest intracellular nitrate pool was found in *G. pacifica* (678.8 ± 143.5 pmol per individual; Table 2) followed closely *C. ovoidea*, having intracellular nitrate concentration of 624.8 pmol/individual (±143.5; three replicates), and *B. subornata* 305.7 ± 259.2 pmol/individual (eight replicates; Table 2). The denitrification rate for *B. subornata* was 39.9 pmol per individual per day (average of three replicates ±16.2; Table 2).

**Table 2:** Average (±SD) intracellular nitrate content per foraminiferal cell and denitrification rates for all tested species.

Species	Nitrate + nitrite pool (pmol/indiv)	Denitrification rate (pmol/ind/day)
*B. subornata*	305.7 ± 259.2 (*N* = 8)	39.9 ± 16.2 (*N* = 3)
*B. striata*	37.4 ± 32.8 (*N* = 2)	NA
*C. ovoidea*	624.8 ± 143.5 (*N* = 3)	NA*
*G. pacifica*	678.8 (*N* = 1)	NA

NA, species not examined for their denitrification capacities. NA*, one specimen of *C. ovoidea* was measured and no denitrification was detected, however, replicate measurements would be required to verify the result.

### Sediment Bacterial Community

**Figure 3:**
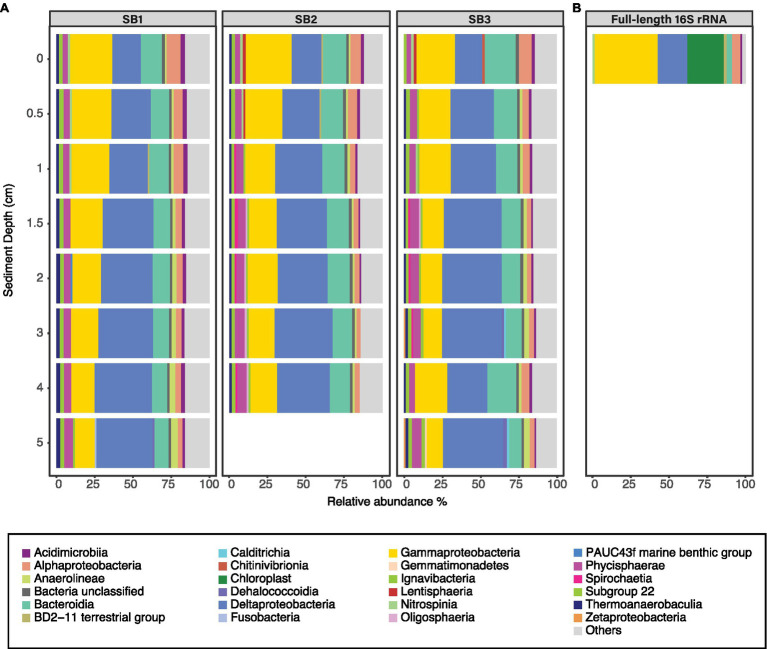
Relative abundance of bacterial classes (RA > 2%) in sediment samples based on Illumina MiSeq **(A)** and full-length **(B)** sequencing of the 16S rRNA gene. The three horizontal facets **(A)** indicate the community in cores SB1, SB2, and SB3. Those bacterial classes that are present <2% RA are grouped in the group “others.”

Sediment bacterial community was dominated by *Deltaproteobacteria* [up to 39.5% relative abundance (RA)], followed by *Gammaproteobacteria* (up to 30% RA) and *Bacteroidia* (up to 20.7% RA; Figure 3A). The class *Deltaproteobacteria* was dominated by the order *Desulfobacterales* (up to 48% RA), *Myxococcales* (up to 24.8% RA), and NB1-j group (up to 24.6% RA). The sediment bacterial community changed with depth (Figure 3A); for example, the class *Deltaproteobacteria* (from average RA 19.5% ±0.8 to 38.5% ±1.3) and *Anaerolinae* (from average RA 1% ±0.1 to 4.3% ±0.3) increased in relative abundance with increasing sediment depths whereas classes *Bacteroidia* (16.6% ±3.5 to 8.9% ±0.8) and *Gammaproteobacteria* (27.8% ±2.3 to 11.8% ±1.6) decreased. The full-length 16S rRNA sequencing of the surface sediment (0–0.5 cm) suggested that bacterial community was dominated by *Gammaproteobacteria* (30.6% RA), followed by *Deltaproteobacteria* (RA 20.4%), Chloroplasts (10.5%), and *Alphaproteobacteria* (9.1%; Figure 3B). PERMANOVA test using the adonis2 function in vegan including two variables (depth and core) confirmed that depth was a significant factor influencing the sediment community variance (*p* = 0.001, *F* = 9.29). The three different cores had similar dominant groups and similar depth-related community changes but displayed some heterogeneity reflected within their varying communities (PERMANOVA *p* = 0.044, *F* = 2.02). The cores had similar Shannon-Wiener (*H*ꞌ) indices, *Hꞌ* ranging from 7.4 ± 0.1 (SB1) to 7.5 ± 0.1 (SB2 and SB3). Core SB1 also had the lowest Pielou’s evenness (*J*ꞌ) index 1.6 ± 0.1, whereas SB3 (1.7 ± 0.4) and SB2 (1.8 ± 0.4) were higher.

### Bacterial Microbiome of Foraminifera: Diversity and Composition

**Figure 4:**
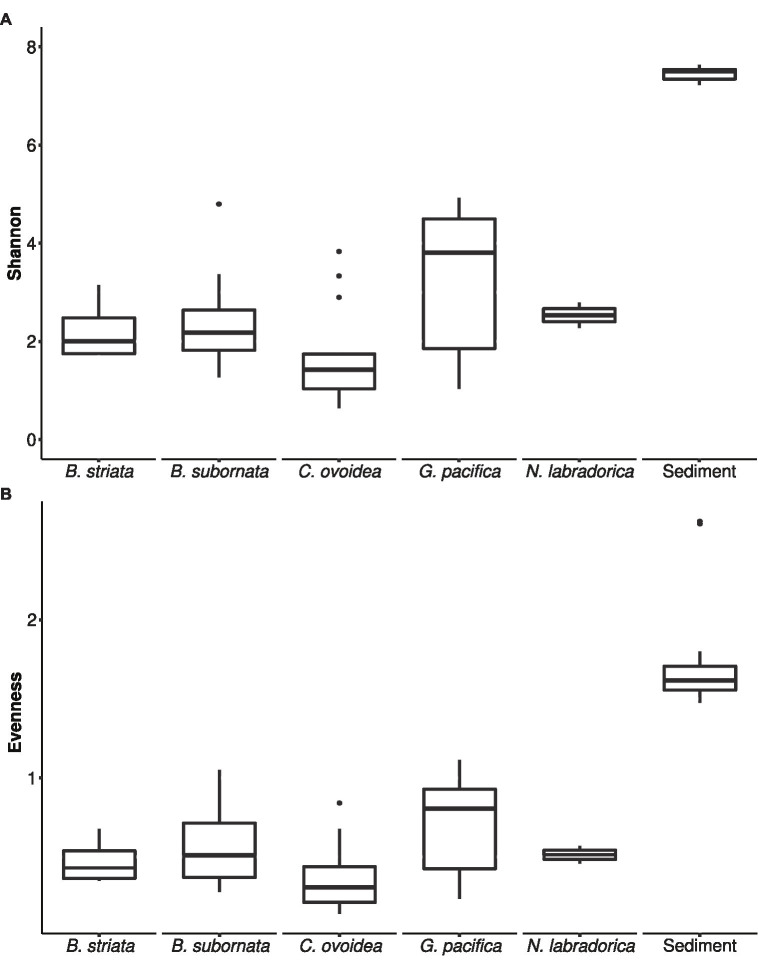
Box and whiskers plot of microbiome alpha-diversity in foraminiferal species and sediment based on Shannon *H*' index **(A)** and Pielou’s evenness index *J*' **(B)**. The lower and upper hinges of the box correspond to the first and third quartiles, the line in between the median, and the whiskers extend to 1.5 times the interquartile range. Values outside this range are represented by black circles.

**Figure 5:**
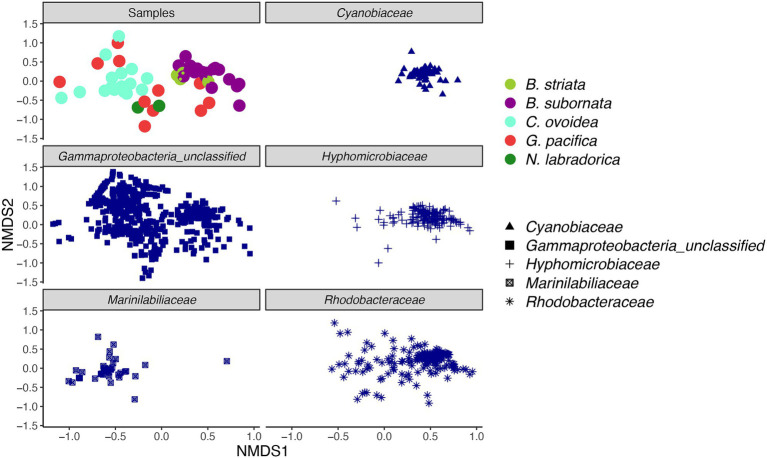
Non-metric multidimensional scaling (nMDS) plot of intracellular bacterial microbiome of foraminiferal species (top left) and five most abundant bacterial families. Foraminiferal species marked in different colors and bacterial families with different shape. nMDS was based on a Bray-Curtis distance (stress 0.198).

Compared to sediment, the alpha diversity (i.e., *Hꞌ* and *J*ꞌ) of the foraminiferal microbiome index was lower (Figure 4). Median *H*ꞌ value for sediment samples was 7.5 (Figure 4), with highest values recorded at sediment depth 1–1.5 (Hꞌ 7.6) and lowest at sediment depth 4–5 cm (Hꞌ 7.2). Median *J*ꞌ for sediment was 1.6 (Figure 4). Among foraminiferal species, highest *H*ꞌ and *J*ꞌ index was recorded for *G. pacifica* with median 4 and 0.8, respectively (Figure 4). Median *H*ꞌ and *J*ꞌ index values for *B. striata* were 2.1 and 0.4 and for *B. subornata* 2.4 and 0.6 (Figure 4), whereas *C. ovoidea* had the lowest *Hꞌ* and *J*ꞌ value of the studied species with medians of 1.5 and 0.3, respectively (Figure 4). PERMANOVA test including three variables (species, sediment depth, and core) indicated that the species was the strongest factor influencing the foraminiferal microbiome (*p* = 0.001, *F* = 5.74), whereas sediment depth (*p* = 0.033, *F* = 1.93) and core (*p* = 0.789, *F* = 0.86) seemed not to have a significant effect on the microbiome composition. As visualized in the nMDS plot, species *C. ovoidea*, *B. subornata*, *B. striata*, and *N. labradorica* were separated into distinguishable group in the ordination space due to intraspecific homogeneity whereas *G. pacifica* specimens depicted more intraspecific variation (Figure 5) as indicated also by the higher *Hꞌ* and *J*ꞌ median (Figure 4). Of the five most abundant bacterial families, *Cyanobiaceae* and *Hyphomicrobiaceae* correlated with *B. striata* and *B. subornata* whereas the family *Marinilabiliaceae* correlated with *C. ovoidea* in the ordination space (Figure 5).

**Figure 6:**
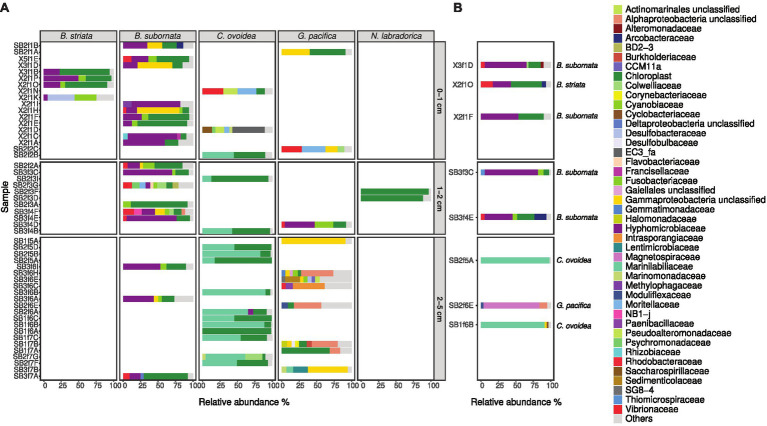
Relative abundance of bacterial families in foraminiferal samples at different sampling depths based on Illumina MiSeq sequencing **(A)** and full-length sequencing **(B)** of the 16S rRNA gene. Bacterial families with RA over 4% **(A)** and 2% **(B)** are shown; taxa present with lower RA are grouped in “Others.”

**Figure 7:**
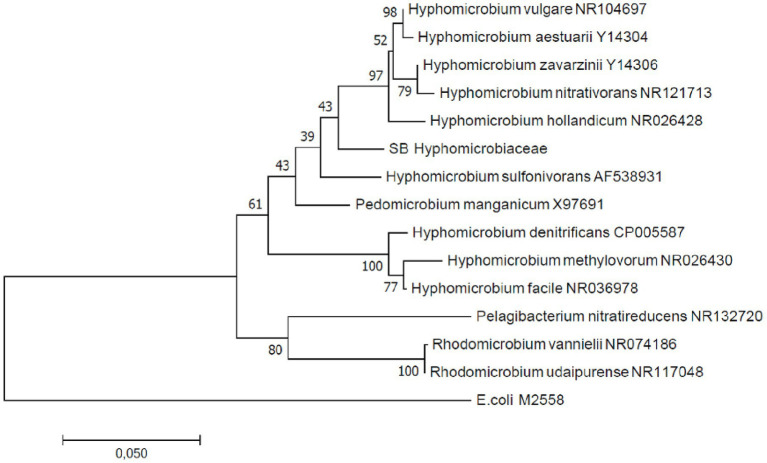
Maximum likelihood phylogenetic tree based on full-length 16S rRNA gene sequences indicating the position of the *Hyphomicrobium* operational taxonomic unit (OTU; SB Hyphomicrobiaceae). GenBank accession numbers are given for the closest relatives after the taxonomic name.

In total, four *B. striata* and 18 *B. subornata* specimens were analyzed. Typically, living *B. subornata* displayed a dark brown, sometimes almost purple color (Supplementary Additional Figure 3). The most abundant bacterial OTUs in both of these species belonged to the family *Hyphomicrobiaceae* [phylum *Alphaproteobacteria*; average across samples 24.9% RA, ±18 (*B. striata*), and 34.8% ±24.5 (*B. subornata*); Figure 6A]. This OTU was present in the majority of the specimens in high relative abundance (up to 71.6% RA), whereas in the surrounding sediments, it had RA < 0.1%, indicating a clear enrichment in the microbiome of these species (Supplementary Additional Table 3). The full-length 16S rRNA gene sequences of four *B. subornata* and one *B. striata* samples confirmed that the most abundant OTU (up to 70% RA) was the same for these two species; a member of the family *Hyphomicrobiaceae* (Figure 6B). In these species, this OTU had the average RA of 45.8% ±17.6 (Supplementary Additional Table 3). Additional BLAST search of the full-length 16S rRNA gene sequence of this OTU indicated that its closest relative was an unclassified member of the *Hyphomicrobiaceae* family with 94.75% ID. Phylogenetic analysis of the *Hyphomicrobiaceae* OTU and its closest relatives along with some other members of representative sequences of *Hyphomicrobiaceae* family revealed, that the Sagami Bay *Hyphomicrobiaceae* OTU groups close to the genera *Hyphomicrobium* (Figure 7).

The total number of *G. pacifica* specimens analyzed was 11. The most abundant OTUs in *G. pacifica* were an unclassified *Gammaproteobacteria* OTU (average RA 18.2% ±30.7) followed by an unclassified *Alphaproteobacteria* OTU (average RA 8.9% ±12.9; Supplementary Additional Table 3; Figure 6A). In the microbiome of a specimen (SB2f6E) of *G. pacifica*, which was subjected to the full-length sequencing of the 16S rRNA gene, 92.8% of the reads belonged to class *Alphaproteobacteria*, of which 85.2% of reads belonged to the family *Magnetospiraceae* (Figure 6B). The most common OTU in the family *Magnetospiraceae* accounted for 45.6% of the reads (Supplementary Additional Table 3).

In total, 16 *C. ovoidea* specimens were analyzed. The most abundant OTU (average across samples 43.4% ±30.4) belonged to group *Bacteroidetes*, family *Marinilabiliaceae* (Figure 6A; Supplementary Additional Table 3). *Bacteroidetes* were also present in the sediment (average RA 13.3% ±2.9; Figure 3A) but dominated by the bacterial families *Flavobacteriaceae*, *Cyclobacteriaceae*, and the group BD2-2 (comprising 23,3, 22,6, and 20.1% of reads in all *Bacteroidetes* OTUs, respectively) whereas only 0.4% of all *Bacteroidetes* reads in the sediment were assigned to the family *Marinilabiliaceae* and <0.01% to the most common *Marinilabiliaceae* OTU in *C. ovoidea* (Supplementary Additional Table 3). Full-length sequencing of the 16S rRNA gene of two *C. ovoidea* specimens confirmed the presence of this bacterial groups, as in average, 83.6% of reads belonged to one OTU of the family *Marinilabiliaceae*, order *Bacteroidales* (Figure 6B; Supplementary Additional Table 3). Additional BLAST search of the full-length 16S rRNA gene sequence indicated that the closest match (93.14% ID) was *Saccharicrinis fermentas* in the family *Marinilabiliaceae*.

### Intracellular Chloroplasts in Foraminifera

**Figure 8:**
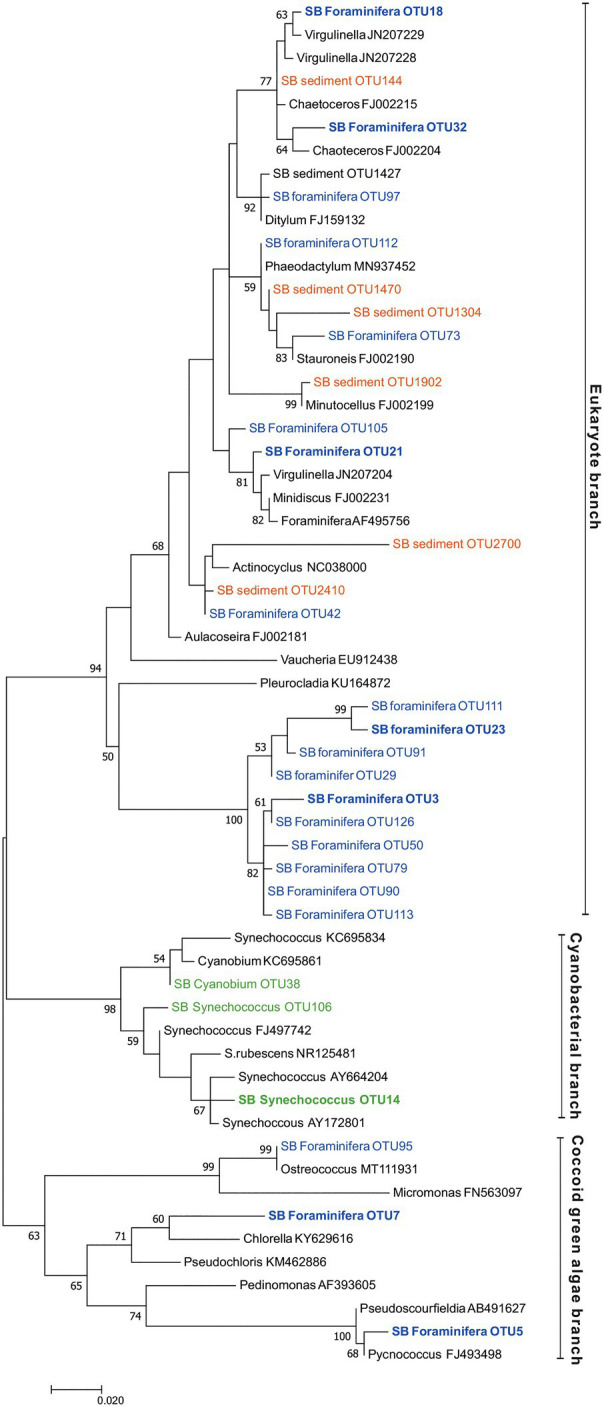
Maximum likelihood tree based on Illumina MiSeq-derived 16S rRNA gene sequences indicating the position of chloroplast OTUs of foraminifera specimens (SB foraminifera OTU, blue color) and sediment samples (SB sediment OTU, orange color) as well as cyanobacterial OTUs (green color). Bootstrap values greater than 50% based on 1000 resamplings are shown at branch nodes. GenBank accession numbers are given for the closest relatives (in black color) after the taxonomic name. The most abundant OTUs are highlighted in bold.

Compared to surrounding sediment, where chloroplasts and *Synechococcales* had a low relative abundance of <1 and <0.1%, respectively (Figure 3A), they were clearly enriched in foraminifera. In *B. striata*, chloroplast OTUs accounted for 42.4% RA (±31.7) in average and OTUs belonging to the order *Synechococcales* had the average relative abundance of 12.3% (±13.3; Figure 6A). The most common chloroplast OTUs in *B. striata* were OTU7 [average RA of all chloroplast reads 40.8% (±36.5)] followed by OTU5 [average RA 22.4% (±24.6; Table 3)]. The closest BLAST match of OTU7 was *Picochlorum* (94.55% ID) and of OTU5 was *Pycnococcus* (98.99% ID), both of which are green algae. In the phylogenetic tree, these OTUs along with OTU95 grouped in the coccoid green algae branch (Figure 8). In *B. subornata* chloroplasts (average 30.2% ±24.2) and *Synechococcales* (average 5.8% ±4.2) were also abundant (Figure 6A). The most common chloroplast OTU (40.4% ±36.5 of all chloroplast reads) in *B. subornata*, was OTU5 followed by OTU7 (average RA of chloroplast reads 31.9% ±19; Table 3). According to the full-length 16S rRNA sequence data, chloroplasts had an average, RA of 26.4% ±14.2 in both *Bulimina* species (Figure 6B).

**Table 3:** The most abundant chloroplast OTUs in foraminifera, including *Cyanobacteria*, based on the average distribution of chloroplast sequence reads (%) per OTU within each species [the most abundant OTU per species is highlighted in blue and SD (±) is presented in parenthesis].

Distribution of reads per chloroplast OTUs within species							
Chloroplast OTU	Closest BLAST match (ID %)	*C. ovoidea*	*B. subornata*	*B. striata*	*G. pacifica*	*N. labradorica*	*Sediment*
SB Foraminifera OTU3	*Vaucheria litorea* EU912438 (86.75)	86.4 (±26.1)	0.6 (±1.9)	0.3 (±0.6)	17 (±32.8)	0 (±0)	0.6 (±0.1)
SB Foraminifera OTU5	*Pycnococcus provasolii* FJ493498 (98.99)	0.4 (±0.7)	40.4 (±23.5)	22.4 (±24.6)	8 (±11.8)	0 (±0)	0 (±0)
SB Foraminifera OTU7	*Picochlorum* sp. “soloecismus” MG552671 (94.55)	0.2 (±0.5)	31.9 (±19)	40.8 (±36.5)	17.5 (±26)	0 (±0)	0 (±0)
SB Synecho-coccus OTU14	*Synechoccous* sp. AY172801 (98.92)	0.1 (±0.2)	12.7 (±8.8)	26 (±34.6)	12.2 (±23.5)	0 (±0)	0 (±0)
SB Foraminifera OTU18	*Virgulinella fragilis* chloroplast JN207229 (98.94)	0.1 (±0.2)	1.7 (±4.8)	0 (±0)	0.5 (±1)	24.8 (±20.2)	0 (±0)
SB Foraminifera OTU21	*Minidiscus trioculatus* FJ002231 (99.22)	0 (±0.1)	0.1 (±0.1)	0 (±0)	2.8 (±6.2)	40.1 (±21.1)	0 (±0)
SB Foraminifera OTU23	*Saccharina latissima* MT151382 (86.12)	0 (±0.1)	0.1 (±0.2)	0 (±0)	16 (±32.4)	0 (±0)	0 (±0)
SB Foraminifera OTU29	*Fucus vesiculosus* MG922855 (88.02)	2.2 (±8.8)	0 (±0)	2.4 (±5)	0.1 (±0.3)	0 (±0)	0.3 (±0)
SB Foraminifera OTU42	Bacterium WHC1-2JQ269270 (98.67)	0 (±0)	0 (±0)	0 (±0)	0 (±0.1)	5 (±7.1)	0 (±0)
SB Foraminifera OTU32	*Chaetoceros* sp. FJ002204 (97.88)	0 (±0.1)	0 (±0)	0 (±0)	0.2 (±0.4)	17.2 (±15.7)	0 (±0)
SB Cyanobium OTU38	*Cyanobium* sp. KC695861 (98.65)	0 (±0.1)	5.3 (±7.3)	3.9 (±5.1)	0.2 (±0.6)	0 (±0)	0 (±0)
SB Foraminifera OTU50	*Vaucheria litorea* EU912438 (87.01)	1 (±1.7)	0 (±0.1)	0.5 (±1.1)	6.7 (±20.1)	0 (±0)	0.1 (±0)
SB Foraminifera OTU73	*Stauroneis simulans* FJ002190 (97.88)	0 (±0)	0.6 (±1.3)	0.6 (±1.2)	0.1 (±0.2)	0 (±0)	0 (±0)
SB Foraminifera OTU79	*Vaucheria litorea* EU912438 (88.31)	0.6 (±0.4)	0 (±0.1)	0 (±0)	0.1 (±0.2)	0 (±0)	0 (±0)
SB Foraminifera OTU90	*Vaucheria litorea* EU912439 (88.05)	0.4 (±0.7)	0 (±0)	0 (±0)	0.1 (±0.2)	0 (±0)	0.1 (±0)
SB Foraminifera OTU91	*Fucus vesiculosus* MG922855 (87.24)	0.3 (±1.1)	0 (±0)	0 (±0.1)	1 (±0)	0 (±0)	0 (±0)
SB Foraminifera OTU95	*Ostreococcus* sp. MT111931 (100)	0 (±0)	0.2 (±0.7)	0.7 (±1.4)	0 (±0)	0 (±0)	0 (±0)
SB Foraminifera OTU97	*Ditylum brightwellii* FJ159132 (99.73)	0 (±0)	0 (±0)	0 (±0)	0 (±0)	1 (±1.4)	5.8 (±1.2)
SB Foraminifera OTU105	*Virgulinella fragilis* chloroplast JN207204 (97.91)	0 (±0)	0 (±0)	0 (±0)	0 (±0)	1.3 (±0.2)	0 (±0)
SB Synecho-coccus OTU106	*Synechococcus* sp. FJ497742 (98.65)	0 (±0)	0.7 (±1.7)	0.2 (±0.5)	0 (±0)	0 (±0)	0 (±0)
SB Foraminifera OTU111	*Saccharina latissima* MT151382 (86.05)	0 (±0)	0 (±0)	0 (±0)	0.8 (±1.7)	0 (±0)	0 (±0)
SB Foraminifera OTU112	*Phaeodactylum tricornutum* MN937452 (99.73)	0 (±0)	0 (±0)	0 (±0)	0.8 (±2.5)	0.7 (±1)	5.7 (±1.2)
SB Foraminifera OTU113	*Vaucheria litorea* EU912439 (87.89)	0.4 (±0.1)	0 (±0)	0 (±0)	0.1 (±0.2)	0 (±0)	0 (±0)
SB Foraminifera OTU126	*Vaucheria litorea* EU912440 (87.56)	5.9 (±23.3)	0 (±0)	0 (±0)	0 (±0)	0 (±0)	0 (±0)

The closest NCBI BLAST match and identity % are also shown, and the average RA % of these chloroplast OTUs of all sediment chloroplasts OTUs.

On average, 92.9% ±4.1 of all intracellular 16S rRNA gene sequences of *N. labradorica* were taxonomically assigned to chloroplasts (Figure 6A). The three most abundant chloroplast OTUs in *N. labradorica* microbiome were OTU21 (average RA of chloroplast reads 40.1% ±21.1), OTU18 (24.8% ±20.2), and OTU32 (17.2% ±15.7; Table 3). These OTUs were not dominant in any other foraminiferal species (RA < 3%), and in the phylogenetic tree, they were found in the eukaryotic branch implying an algal instead of cyanobacterial origin for these OTUs (Figure 8).

Chloroplasts were found to be abundant in *G. pacifica* were they accounted for 13.3% ±21.6 in average of the intracellular OTUs (Figure 6A). Most common chloroplast in *G. pacifica* was OTU7 (average RA 17.5% ±26) followed by OTU3, OTU23, OTU14, and OTU5 (Table 3).

In *C. ovoidea*, chloroplasts accounted on average for 38.7% ±30.8 of the intracellular 16S rRNA gene derived OTUs of (Figure 6). Of these OTUs, on average, 86.4% ±26.1 belonged to a single OTU, OTU3 (Table 3). Additional BLAST search indicated that the closest match of OTU3 (86.75% ID, excluding uncultured/environmental samples) was *Vaucheria litorea* chloroplast, which is a species of yellow-green algae (ID 86.75; Table 3). In sediments and other foraminiferal species, except for some *G. pacifica* specimens, this OTU was present in very low numbers (Table 3). In the phylogenetic tree (Figure 8), OTU3 grouped together with other chloroplasts found in lower abundance within the foraminiferal samples, in particular *C. ovoidea*. Conversely, the full-length 16S rRNA gene data contained only a low relative abundance (<1%) of chloroplasts in *C. ovoidea* specimens (Figure 6B).

## Discussion

### Differences of Bacterial Community Structure in Sediments and in Foraminiferal Microbiome

According to our results, the microbiome of the deep-sea benthic foraminifera differed from the surrounding sediment bacterial community. For example, all sediment cores had relatively similar alpha diversity values, whereas foraminiferal species displayed higher degree of intra- and inter-specific variation, as for example, the median Shannon diversity index for *G. pacifica* was 4 but for *C. ovoidea* only 1.5. Previously, shallow-water foraminifera from intertidal areas have also been observed to have a microbiome, which is distinct from the surrounding sediment communities (Salonen et al., 2019). In contrast, some larger benthic foraminifera have been discovered to have a site-specific microbiome (Prazeres et al., 2017). In this study, no spatial analyses were carried out and any depth-related changes are likely to reflect the associated changes in pore water redox chemistry and associated changes in sediment bacterial community. Instead, our results clearly demonstrated that in deep-sea foraminifera, the driving factor of the variability of the foraminiferal microbiome was solely the species (*p* = 0.001). Compared to sediments, the intracellular microbiome of foraminifera was less diverse, and typically dominated by few key bacterial groups, with the exception of *Nonionella labradorica*, where bacterial OTUs had the RA of <10% as their microbiome was quasi-solely dominated by chloroplasts. In general, bacterial groups abundant in sediments, such as *Deltaproteobacteria*, comprised only a small part of the foraminiferal microbiomes. Bacteria could represent a food source for foraminifera, however, bacterivory is not likely to be the preferred trophic strategy of these species or benthic foraminifera in general; instead, it is likely to occur randomly during deposit-feeding (van Oevelen et al., 2006; Nomaki et al., 2008). If the foraminiferal microbiome would primarily result from bacterivory or deposit-feeding, it would be expected to closely resemble the sediment bacterial community, which was not the case here. Instead, specific bacterial taxa, typically rare in sediments, were found to constitute a significant proportion of the foraminiferal microbiomes, suggesting that there are species-characteristic, selective mechanisms, and potential endobiotic interactions influencing the bacterial composition of the foraminiferal microbiome. The successfully amplification of long DNA fragments (>1,400 bp) of the key intracellular bacterial groups from single foraminiferal cells, further corroborates that bacterial DNA was relatively intact and not degraded due to digestion.

### Species-Characteristic Foraminiferal Bacterial Microbiome

Endobiotic relationships with bacteria could provide the foraminiferal host, an ecological advantage in the hypoxic sediments, by for example providing the host with alternative respiration and/or carbon and nitrogen assimilation pathways (Bernhard et al., 2011; Tsuchiya et al., 2015). In *B. striata* and *B. subornata*, the full-length 16S rRNA gene sequence reads confirmed the presence of associated bacterial family *Hyphomicrobiaceae*, also suggested by the shorter sequence data. The family *Hyphomicrobiaceae* usually displays a variety of chemo-organotrophic metabolic pathways ranging from sulfide oxidation and metal cycling to denitrification (Stein et al., 2001; Osaka et al., 2006; Gliesche et al., 2015). Some species belonging to this bacterial family have been reported to have a substantial role in the marine nitrogen cycle (Anderson et al., 2011). A key adaptation of foraminifera to hypoxic sediments is the ability to collect nitrate and denitrify (Risgaard-Petersen et al., 2006), a respiration pathway which has also been confirmed for *B. subornata* (Table 2). Some foraminiferal species are able to perform denitrification themselves (Risgaard-Petersen et al., 2006; Woehle et al., 2018), however, it is possible that some species rely on denitrifying endobionts (Bernhard et al., 2011). For example, if *B. subornata* does not perform denitrification itself, the endobiotic bacterial family *Hyphomicrobiaceae* could be involved in this process as members of this bacterial family have denitrifying capabilities (Osaka et al., 2006). Alternatively, the family *Hyphomicrobiaceae* could be involved in metal, such as manganese, oxidation (Stein et al., 2001), or play another yet unknown biochemical role in this foraminiferal species.

*Chilostomella ovoidea* is a deep-infaunal species, typically found in anoxic sediments in 3–5 cm sediment depth (Corliss, 1985; Ohga and Kitazato, 1997; Nomaki et al., 2005). Relatively high intracellular nitrate content has been measured in *C. ovoidea* (Table 2; Piña-Ochoa et al., 2009), however, further experiments would be needed to investigate the potential nitrate reduction through denitrification in this species (Table 2). Here, *C. ovoidea* specimens displayed a very distinct low-diversity microbiome, where a bacterial taxonomic group belonging to family *Marinilabiliaceae*, class *Bacteroidetes*, was the most abundant. In sediments, the bacterial family *Marinilabiliaceae* was rare compared to other *Bacteroidetes* families, and it was not abundant in the microbiome of any other foraminiferal species. Moreover, as the sediment sample used for bacterial DNA extractions was intact, it may have contained also a low number of foraminifera. Therefore, it cannot be excluded that the sediment signal of *Marinilabiliaceae* could be also derived from foraminifera. The group *Bacteroidetes* is commonly found in marine sediments including in the deep-sea, where they typically grow anaerobically, degrading particulate matter such as proteins (Fernández-Gómez et al., 2013). In this class, bacteria belonging to the family *Marinilabiliaceae* are Gram-negative, motile rods that display saccharolytic growth, and some genera have been isolated previously from marine mud containing decaying algae (Ludwig et al., 2015). The species *Saccharicrinis fermentas*, to which the *Marinilabiliaceae* group was closest related according to BLAST search (ID 93.14%), is a facultatively anaerobic chemo-organotroph that does not have denitrifying capacities (Yang et al., 2014).

*Globobulimina pacifica* is also a deep-infaunal species known to thrive in low-oxygen conditions (e.g., Corliss, 1985; Jorissen et al., 1995; Fontanier et al., 2002). This species can collect nitrate and denitrify (Table 2; Langlet et al., 2020). In previous studies, genus *Globobulimina* has been shown to perform complete denitrification itself (Woehle et al., 2018) and contain intracellular bacteria only in low abundance, suggesting a lack of bacterial endobionts (Risgaard-Petersen et al., 2006). Here, bacterial signal was retrieved from *G. pacifica*, but in contrast to other foraminiferal species, the microbiome composition seemed to be more heterogenous, which was also reflected in its Shannon diversity, ranging from 1.1 to 5.1 between specimens. Yet, *G. pacifica* microbiome composition was also significantly different from the surrounding sediment community. Four specimens of *G. pacifica* collected from the deeper (2–5 cm) sediment layers contained abundant *Alphaproteobacterial* OTUs, which in the specimen (SB26E) was confirmed by the analyses of the full-length 16S rRNA gene to belong to bacteria family *Magnetospiracea*. This family includes metal reducing magnetotactic and microaerophilic/anaerobic bacteria that have been suggested to have a role in Fe^3+^ mobilisation and bioavailability (Schüler and Frankel, 1999; Molari et al., 2020). Previously, magnetotactic bacteria have been observed to form symbiotic relationships with other protists, *Euglenozoa*, potentially helping the host to navigate in anoxic sediments toward optimal chemical niches (Monteil et al., 2019). The genus *Globobulimina* has been observed to distribute in sediments tightly linked with Fe^2+^ oxidation zone, which could be an indication of a specialized lifestyle and a potential trophic or symbiotic relationship with chemolithoautotrophic prokaryotes (Fontanier et al., 2005). However, due to variability in the microbiome composition and limited full-length sequence data, further research is required to verify the amount and potential meaning of these bacterial groups in *G. pacifica*. Potentially, the variability observed in the microbiome could also reflect the trophic strategy of this species. In previous experiments from Sagami Bay, *Globobulimina affinis* showed a selective uptake of algae, suggesting a preference for phytodetritus as a primary food source when available (Nomaki et al., 2006), although the sterol compositions of *G. affinis* and surrounding sediments suggest that in general it possesses a deposit-feeding strategy (Nomaki et al., 2009). Potentially, *G. pacifica* has a more opportunistic lifestyle, which might include various ecological behaviors, leading to the variability in the microbiome.

To resolve the ecological significance and potential function of intracellular bacteria, such as of *Marinilabiliaceae* for *C. ovoidea* and *Hyphomicrobiacea* for *B. subornata*, more data are required on the activity and intracellular location of these bacterial groups. In addition, the method of symbiont transmission and acquisition for benthic foraminifera remains to be resolved. Previously, it has been suggested that some foraminiferal species may retrieve their putative endobionts from the surrounding environment (Bird et al., 2017; Salonen et al., 2019). In the case of *C. ovoidea* and both *Bulimina* species, the putatively endobiotic bacterial groups were also present in the surrounding sediment, but in very low abundance (<0.1% RA). Alternatively, foraminifera could acquire the endobionts *via* asexual reproduction and co-occurring vertical transmission of endobiotic bacteria (Tsuchiya et al., 2009, 2015; Takagi et al., 2020).

Although depth was not a significant parameter explaining the foraminiferal microbiome composition, in this study, we observed that two *C. ovoidea* specimens (X2f1N and X2f1D) retrieved from the surface sediment (0–1 cm depth) had a distinct microbiome compared to the specimen SB2Bf2B (retrieved from 0.5–1 cm sediment depth) and all specimens from deeper sediment depths. Two surface specimens also had higher Shannon diversity indices (median 3.3 and 3.9) compared to deeper specimens (median 1.4 below 1 cm depth), and interestingly, the surface specimens lacked the anaerobic bacterial group *Marinilabiliaceae*, which was dominant in the other specimens. However, due to the low number of specimens recovered from certain depth intervals, we are not able to drive secure conclusions. Future studies may resolve the possibility that vertical distribution and availability of oxygen could be linked to the composition of the microbiome of *C. ovoidea* – perhaps either by providing the suitable environmental conditions for its sustenance or the putative endobionts themselves. In addition, further studies are needed to resolve the stability of the foraminiferal microbiome in temporal and spatial scale.

### Intracellular Chloroplasts

In the surrounding sediment, chloroplasts accounted for less than 1% RA of all OTUs at all sampling depths whereas majority of foraminiferal specimens of all studied species had a significantly higher intracellular chloroplast OTUs RA (ranging from 92.9 ± 6% RA in *N. labradorica* to 15.3 ± 23% in *G. pacifica*). In sediment, the chloroplasts originated mainly from diatoms, with the most common OTU (SB Sediment OUT 144; Figure 8) that accounted 59.4 ± 12.8% of the chloroplast reads assigned to *Chaoteceros calcitrans* (BLAST ID 99.73). According to the phylogenetic analysis, the chloroplast OTUs in foraminifera originated from several sources, of which some were bacterial and some eukaryote-related (Figure 8). In sediments, the most common chloroplasts originated from eukaryote, namely diatom sources. Some chloroplast OTUs were tightly linked to a specific foraminiferal species (Table 3), implying species-characteristic preferences in terms of chloroplast sources.

A large abundance of chloroplasts was observed in the intracellular microbiomes of the two *Nonionella labradorica* specimens where they accounted for >90% of total OTU abundance. Although chloroplasts strongly dominated the microbiome of *N. labradorica*, relatively high *H*ꞌ and *J*ꞌ indices (2.4, 2.9, and 0.5, 0.6, respectively) can be explained by the variability in the chloroplast OTUs (Table 3) and the rare (bacteria had the average RA 7% ±5.7) yet diverse intracellular bacterial community (median *H*ꞌ 5.5 excluding chloroplasts). Based on the phylogenetic analysis, the most common chloroplasts of *N. labradorica* originated from eukaryote sources (mainly diatoms), which were common also in the surrounding sediment (Figure 8). The intracellular chloroplast OTUs of foraminifera may be linked to herbivorous feeding behavior as feeding experiments have shown that many benthic foraminiferal species ingest algae (e.g., Moodley et al., 2000; Nomaki et al., 2005, 2006). If the chloroplasts retained by the *N. labradorica* are used for nutrition, their high enrichment in the cytoplasm could indicate that part of the survival strategy of this species is to store food, in this case chloroplasts, intracellularly. Alternatively, foraminifera living in the photic zone have a well-documented ability to collect and retain chloroplasts, which they then utilize in a mixotrophic life strategy called kleptoplasty (Lee et al., 1989; Bernhard and Bowser, 1999; Cesbron et al., 2017). Curiously, some deep-sea foraminifera within the genus of *Nonionella* have been found to retain active chloroplasts in the non-photic zone, even at depths up to 600 m (Bernhard and Bowser, 1999; Jauffrais et al., 2018). Although the exact reason why these specimens retain chloroplasts in the non-photic zone remains unclear, it has been suggested that they may be used for ammonium or sulfate assimilation pathways similarly to non-photosymbiontic algae (Grzymski et al., 2002; Jauffrais et al., 2018).

Previously, *B. subornata* and *B. striata* have shown δ^13^C values typical for phytophagous species, implying a preference for algal diet (Nomaki et al., 2008). Here, the phylogenetic analysis of the most common chloroplast OTUs inside *B. subornata* and *B. striata* revealed that they were closely associated to *Pycnococcus provasolii* (98.99% ID) and *Picochlorum* sp. (94.55% ID; Table 3). Both of these species are small, coccoid green algae commonly found in marine pelagic environments (Campbell et al., 1994; Henley et al., 2004), some of them able to accumulate high amounts of lipids (Unkefer et al., 2017). As the most common chloroplasts in the sediment samples belonged to diatoms, the chloroplast composition of *B. subornata* and *B. striata* suggests that these species could potentially favor these green algae as a nutrition source over diatoms and selectively ingest them. Therefore, the large intracellular abundance of these chloroplasts (>30% RA, on average) in these species is most likely reflecting their phytodetrivorous trophic preference, which may also be linked to their shallow infaunal distribution in sediment.

Unlike, in other studied species, the chloroplast sequences detected in *C. ovoidea* was highly specific and more than 85% of them belonged to only one OTU (OTU 3). The closest BLAST hit (86.8% ID) of this OTU was a filamentous green-yellow algae *Vaucheria litorea*, the chloroplasts of which are used by the sea slug *Elysia chlorotica* as intracellular photosymbionts (Mujer et al., 1996). However, this closest match was of low identity (only 86.8%) corroborated by the phylogenetic analysis which clustered this chloroplast in a phylogenetically separate branch rather than close to *V. litorea*. This is a well-supported branch (100% support based on 1,000 bootstrap replicates) unique to the foraminiferal specimens of this study. This foraminiferal branch was part of the eukaryote branch of the phylogenetic tree (Figure 8), neighboring with chloroplasts from algal origins (*Vaucheria* and *Pleurocladia*), suggesting a eukaryotic origin of this OTU rather than a cyanobacterial one. Chloroplast OTUs in this branch (>90% of reads comprising this branch) were associated with *C. ovoidea* (except OTUs 23 and 111, >98% associated with *G. pacifica*), implying that similar microhabitat preference of *C. ovoidea* might explain their association to these chloroplast OTUs.

Unlike those foraminiferal species that prefer surface sediment as their microhabitat, *C. ovoidea* does not usually display strong seasonality or response to phytodetritus input, and thus it has been thought to be a slow-growing species with a preference for more refractory organic matters (Ohga and Kitazato, 1997; Nomaki et al., 2005). In feeding experiments, *C. ovoidea* has shown almost no or random ingestion of algae, DOM, and bacteria, suggesting either non-selective deposit-feeding or that their food preferences and life strategy are very specific and still unknown, and thus not well-captured in experimental studies (Nomaki et al., 2005, 2006). Potentially, the unique chloroplast OTUs in *C. ovoidea* could be resulting from a very specific type of selective herbivory that was uncaptured in feeding studies where a different type of algae was used. However, the OTUs of the chloroplast branch associated with *C. ovoidea* were extremely rare in sediments (0.001% RA), challenging the plausibility of the uptake of these chloroplasts directly from the surroundings. Alternatively, this OTU could belong to an algal symbiont, which the foraminifera potentially transfer asexually, as suggested for some foraminiferal species (Tsuchiya et al., 2015). Recently, the ultrastructure of *C. ovoidea* was found to contain abundant chitinous structures that are not commonly observed in other foraminifera, highlighting that this particular species has unique ecological characteristics (Nomaki et al., 2020). The function and origin of these structures are still unknown. They may be synthetized either by the foraminifera itself or alternatively linked to specific type of feeding or symbiosis (Nomaki et al., 2020). If the origin of the chitinous structures is algal, the distinct chloroplast signal in *C. ovoidea* could be linked to the same phenomenon. However, in contrary to chloroplast reads in both *Bulimina* species, the chloroplast OTUs of two *C. ovoidea* (SB1 6B and SB2 5A) did not amplify with full length 16S rRNA gene sequencing, although the specimens seemed to contain intracellular chloroplasts according to the shorter amplicon data. This could, for example, be due to potential degradation of the DNA, which would preclude the extraction and amplification of long DNA fragments. However, as the universal bacterial 16S rRNA gene amplicons provide only limited information on intracellular chloroplasts, further studies using for example 18S rRNA gene metabarcoding (Jauffrais et al., 2018; Chronopoulou et al., 2019) or genomic approaches would be advisable in elucidating the source and meaning of this chloroplast OTU for *C. ovoidea.*

## Conclusion

16S rRNA gene metabarcoding provides a powerful tool for investigating and elucidating the microbiomes of even unicellular eukaryotes, such as the foraminifera. Here, deep-sea foraminifera, representing various microhabitats, were found to harbor microbiomes that clearly differed from one species to another in terms of the relative abundance of the dominant intracellular bacteria and chloroplasts, as well as diversity and degree of intra-specific variation. Based on these observations, we were able to identify two bacterial groups that were strongly associated to specific foraminiferal species; the *Marinilabiliaceae* bacterial group for *C. ovoidea* and the *Hyphomicrobiaceae* for *B. subornata* and *B. striata*. The high relative abundance and consistent occurrence of these bacteria in these species could insinuate a stable and putatively endobiotic relationship, therefore calling for further research focusing on these bacterial groups. In addition, the origins of chloroplasts differed between species, most likely reflecting the divergent ecological and trophic strategies of the studied species.

## Data Availability Statement

The datasets presented in this study can be found in online repositories. The names of the repositories and accession numbers can be found at: https://www.ncbi.nlm.nih.gov/bioproject/PRJNA679187/.

## Author Contributions

KK conceived the study. IS, P-MC, KK, and HN carried out the 2017 sampling campaign and sample processing in the field. DL carried out the oxygen profiling and the foraminiferal nitrate pool and denitrification measurements. IS, P-MC, HN, DL, and KK identified and picked the living foraminifera. HN carried out the 2019 sampling campaign including oxygen profiling and porewater nutrient analysis. IS carried out the molecular laboratory work including DNA extractions and amplifications, completed bioinformatical, statistical, and phylogenetic analysis of 16S rRNA gene sequence data, and drafted the manuscript. P-MC assisted with the phylogenetic and bioinformatical analysis. All authors contributed to the interpretation of the results and the final version of the manuscript.

## Conflict of Interest

The authors declare that the research was conducted in the absence of any commercial or financial relationships that could be construed as a potential conflict of interest.

## Publisher’s Note

All claims expressed in this article are solely those of the authors and do not necessarily represent those of their affiliated organizations, or those of the publisher, the editors and the reviewers. Any product that may be evaluated in this article, or claim that may be made by its manufacturer, is not guaranteed or endorsed by the publisher.
