# Supplementary material for: 16S rRNA Gene Metabarcoding Indicates Species-Characteristic Microbiomes in Deep-Sea Benthic Foraminifera
Source: Front Microbiol. 2021 Jul 27;12:694406. doi: 10.3389/fmicb.2021.694406 (PMC8353385; doi:10.3389/fmicb.2021.694406)
Supplement: Supplementary file 1 [file Data_Sheet_1.PDF]

Additional files

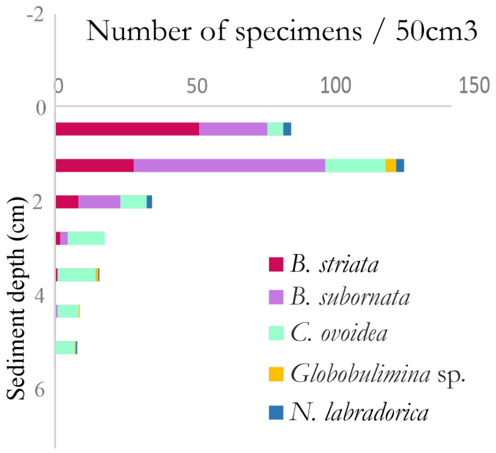

Additional figure 1. Vertical distribution of foraminifera (*B. striata*, *B. subornata*, *C. ovoidea*, *Globobulimina* sp. and *N. labradorica*) in sediments down to 5 cm sediment depth. For this measurement, two cores (SB4 and SB5) were sliced with 0.5 cm intervals down to two cm depth and then with one cm intervals down to five cm depth. Samples were fixed with ethanol (99%) and Rose Bengal (1g/L). Samples were sieved over 63 µm and oven dried. Prior to picking the sample was dry sieved over 150 µm mesh to separate the larger foraminifera, the size fraction also used in the molecular study, which were subsequently counted and identified.

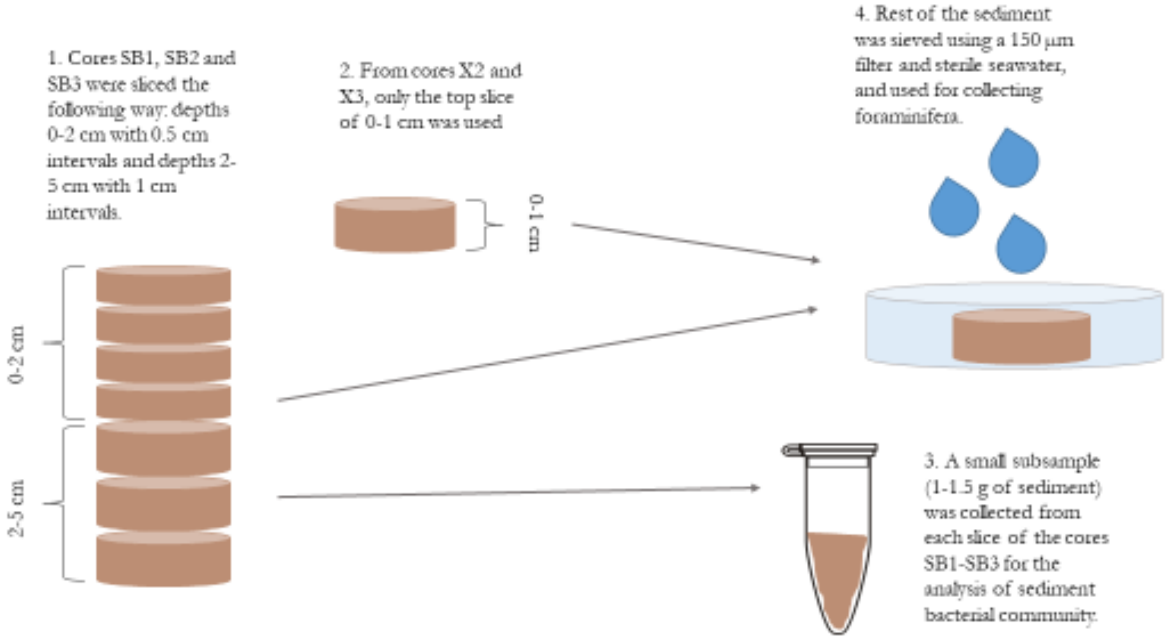

Additional figure 2. Processing of the sediment cores for foraminiferal and sediment samples.

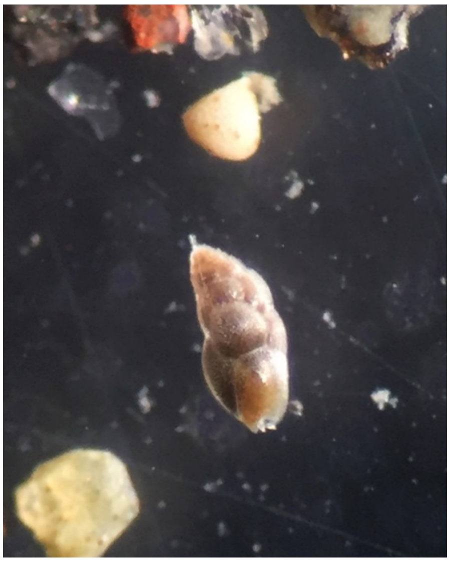

Additional figure 3. Light microscopy image of *B. subornata*

Additional table 1. Number of foraminiferal specimens during the extraction procedure

Step	Initial number of specimens	Specimens lost %
**Picking and cleaning**	99	40.4
**Amplification**	59	15.3
**Sequencing**	50	0

Additional table 2. Number of OTUs and reads per sample.

Foraminifera			Sediment		
Sample	OTUs	Reads	Sample	OTUs	Reads
SB1f5A	338	87812	SB1s1	5998	91319
SB1f6A	252	63691	SB1s2	7336	98325
SB1f6B	191	58662	SB1s3	7003	81217
SB1f6C	243	73075	SB1s4	6930	73953
SB1f7A	376	35540	SB1s5	7016	87711
SB1f7B	348	4748	SB1s6	6692	78020
SB1f7C	185	10269	SB1s7	6528	94900
SB2f1A	303	90667	SB1s8	6489	89172
SB2f1B	618	76426	SB2s1	5843	74266
SB2f2A	760	99739	SB2s2	6459	54050
SB2f2B	571	75753	SB2s3	7634	88786
SB2f2C	1815	78428	SB2s4	7254	76731
SB2f3A	511	118639	SB2s5	7792	118381
SB2f3D	306	45188	SB2s6	6163	68918
SB2f3F	582	122325	SB2s7	7262	108160
SB2f3G	1679	69894	SB3s1	5929	75035
SB2f3I	361	38397	SB3s2	7133	79638
SB2f5A	246	147479	SB3s3	7088	66387
SB2f5B	219	51182	SB3s4	6948	75330
SB2f5D	185	36228	SB3s5	6767	75600
SB2f6A	212	20982	SB3s6	6380	87477
SB2f6E	393	13897	SB3s7	7156	74001
SB2f7F	301	42082	SB3s8	5060	66115
SB2f7G	1330	93277			
SB3f3C	518	104947			
SB3f4B	509	115195			
SB3f4D	270	37315			
SB3f4E	157	42761			
SB3f4F	41	717			
SB3f6A	198	3063			
SB3f6B	55	9428			
SB3f6C	143	1134			
SB3f6E	143	7267			
SB3f6H	244	9996			
SB3f6I	173	12182			
SB3f7A	284	43721			
SB3f7B	40	6493			
X2f1A	680	65339			
X2f1C	285	21613			
X2f1D	1204	87212			
X2f1E	643	58769			
X2f1F	717	151293			
X2f1H	499	56003			
X2f1I	402	47795			
X2f1K	468	65098			
X2f1N	466	10959			
X2f1O	623	64007			
X2f1P	337	35588			
X3f1B	364	72495			
X3f1D	657	33220			
X5f1E	666	116723			

Additional table 3. Average relative abundance of most common bacterial OTUs in foraminifera and their RA in sediment (average including all depths) according to short-read and full-length sequencing of the 16S rRNA gene.

	Bacterial OTU	*B. subornata*	*B. striata*	*N. labradorica*	*C. ovoidea*	*G. pacifica*	Sediment
**V1-V3 region of 16S rRNA**	*Alphaproteobacteria; Rhizobiales; Hyphomicrobiaceae*	34.8 ± 24.5	24.9 ± 18	0 ± 0	0.6 ± 1.8	4 ± 12.8	0.1 ± 0
	Bacteroidetes; Bacteroidales; Marinilabiliaceae	0.1 ± 0.4	0.1 ± 0.1	0 ± 0	43.4 ± 30.4	1.1 ± 2.9	0.003 ± 0
	Gammaproteobacteria; Unclassified	0 ± 0.1	0 ± 0	0 ± 0	0 ± 0	18.2 ± 30.7	0 ± 0
	Cyanobacteria; Synechococcus	4.5 ± 4.1	10.1 ± 11.8	0 ± 0	0 ± 0	3.1 ± 7.8	0.01 ± 0
	Gammaproteobacteria; Unclassified	8.7 ± 17.6	0.4 ± 0.9	0 ± 0	0 ± 0	0 ± 0	0.01 ± 0
	Alphaproteobacteria; Unclassified	0.2 ± 0.8	0 ± 0	0 ± 0	0 ± 0	8.9 ± 12.9	0.01 ± 0
**Full-length 16S rRNA**	*Alphaproteobacteria; Rhizobiales; Hyphomicrobiaceae*	51.4 ± 14.3	23.4	NA	0	0	0.1
	Bacteroidetes; Bacteroidales; Marinilabiliaceae	0	0	NA	83.6 ± 2.3	0	0
	Alphaproteobacteria; Rhodospirillales; Magnetospiraceae	0	0	NA	0	45.6	0
	Epsilonproteobacteria; Campylobacteria; Arcobacter	3.8 ± 7.6	1.4	NA	0	0	< 0.1
	Cyanobacteria; Synechococcus	3.7 ± 2.6	5.3	NA	0	0	0
